# Validation of an AI Method for Automated Lymphoma Metabolic Tumor Volume Segmentation Using a Public Benchmark PET/CT Dataset

**DOI:** 10.2967/jnumed.125.271605

**Published:** 2026-06

**Authors:** May Sadik, Måns Larsson, Olof Enqvist, Lars Edenbrandt, Elin Trägårdh

**Affiliations:** 1 Department of Molecular and Clinical Medicine, Sahlgrenska University Hospital, Sahlgrenska Academy at University of Gothenburg, Gothenburg, Sweden;; 2 Eigenvision AB, Lund, Sweden;; 3 Department of Electrical Engineering, Chalmers University of Technology, Gothenburg, Sweden;; 4 Department of Clinical Physiology and Nuclear Medicine, Skåne University Hospital, Malmö, Sweden; and; 5 Department of Translational Medicine and Wallenberg Center for Molecular Medicine, Lund University, Malmö, Sweden

**Keywords:** lymphoma, total metabolic tumor volume, artificial intelligence, deep learning, benchmark dataset

## Abstract

The aim of this study was to evaluate the performance of an artificial intelligence (AI)–based method for automated segmentation of total metabolic tumor volume (TMTV) in ^18^F-FDG PET/CT scans of patients with lymphoma, using an independent, publicly available benchmark dataset curated and segmented by expert readers in a previously published study. **Methods:** The AI model, based on a 3-dimensional U-Net architecture implemented in MONAI (the medical open-source network for AI framework), was trained on 1,500 ^18^F-FDG PET/CT scans of patients with lymphoma. It was tested on a benchmark dataset comprising 60 baseline scans (20 each of follicular lymphoma, Hodgkin lymphoma, and diffuse large B-cell lymphoma), each segmented by 3 or 4 nuclear medicine physicians using an SUV threshold of 4. Agreement between AI-derived and benchmark TMTVs was assessed using Bland–Altman analysis, with acceptable deviation defined as within 10% or 10 cm^3^, consistent with interreader variability reported in the benchmark study. **Results:** In 50 (83%) of the 60 benchmark cases, AI-derived TMTVs were within 10% or 10 cm^3^ of the benchmark reference. In 4 of the remaining 10 cases, AI-derived results were within the same margin of at least 1 of the expert readers, indicating partial concordance. **Conclusion:** The AI-based method achieved high concordance with expert-derived TMTVs in a standardized benchmark setting. The findings demonstrate that the AI model performs comparably to human experts in most cases, even in an externally curated dataset deliberately enriched with challenging cases by its original authors. The AI model’s ability to produce accurate, reproducible segmentations without user interaction could significantly reduce manual workload and interreader variability in lymphoma imaging. However, human supervision is required to minimize errors.

## Visual Abstract

**Figure:**
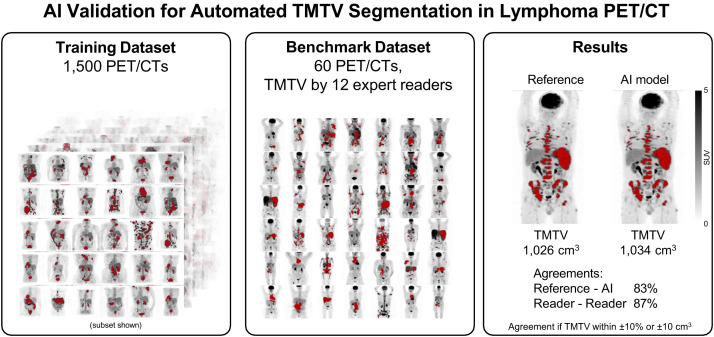

Accurate quantification of total metabolic tumor volume (TMTV) from baseline ^18^F-FDG PET/CT imaging has emerged as a powerful prognostic biomarker in lymphoma, offering superior risk stratification compared with traditional staging methods and clinical scores (1–7). TMTV has demonstrated predictive value across multiple lymphoma subtypes, including Hodgkin lymphoma, follicular lymphoma, and diffuse large B-cell lymphoma, and is increasingly being considered for use in guiding treatment decisions and monitoring therapeutic response (8–11).

Despite its clinical relevance, widespread adoption of TMTV in routine practice and clinical trials has been hindered by the lack of standardized segmentation methods. Variability in lesion delineation, driven by differences in software tools, thresholding strategies, and reader interpretation, can lead to significant discrepancies in measured TMTVs, complicating comparisons across studies and limiting the utility of TMTV as a reproducible biomarker (12–19). Recognizing this challenge, an international consortium recently developed and published a benchmark dataset comprising 60 baseline ^18^F-FDG PET/CT scans of patients with lymphoma, segmented by 12 nuclear medicine experts, with each case processed by 3 or 4 readers (20). This benchmark provides a valuable reference for the evaluation and validation of segmentation methods, with the goal of harmonizing TMTV measurements across institutions and software platforms.

In previous work, we developed an artificial intelligence (AI)–based method for automated segmentation of TMTV in ^18^F-FDG PET/CT images of patients with lymphoma (21–23). Our approach leverages deep learning to identify and delineate metabolically active tumor regions with no user interaction, aiming to improve reproducibility, reduce manual editing, and accelerate clinical workflows. In this study, we build on these previous studies and have expanded the training cohort from 156 to 1,500 patients and included images from more hospitals. We evaluated the performance of our AI-based segmentation method using the publicly available benchmark dataset described by Boellaard et al. (20)—a proposed international benchmark dataset intended to standardize TMTV measurements in lymphoma. We assessed the accuracy of our method by comparing our results to expert-derived reference TMTVs.

## MATERIALS AND METHODS

### Patients

The training set of 1,500 ^18^F-FDG PET/CT studies was obtained from 2 hospitals and 2 databases from the Cancer Imaging Archive (FDG PET/CT lesions (24) and CALGB50303 (25)). Patient characteristics are shown in Table 1.

**TABLE 1.** Patient Characteristics

	Dataset			
Characteristic	Skåne UH	AutoPET	Sahlgrenska UH	CALGB
*n*	624	503	258	115
Sex				
Female	251	241	118	—
Male	373	262	140	—
Age (y)	52 (17–88)	55 (11–85)	43 (10–85)	—
Lymphoma type				
Hodgkin	281	—	177	—
DLBCL	322	—	60	115
Follicular	6	—	2	—
Other	15	138	19	—
Negative controls	—	365	—	—

UH = University Hospital; DLBCL = diffuse large B-cell lymphoma. Data are presented as number; age is presented as mean with range in parentheses.

The study was approved by the ethics committees at the University of Gothenburg and Lund University or by the Swedish Ethical Review Authority. The need for written informed consent was waived for patients from Gothenburg University (2019-01274 and 2024-08225-02), whereas all patients from Skåne University Hospital provided written informed consent before entering the following studies: #2016/417, #2018/117, #2018/753, and #2021-05734-02. This study was performed in accordance with the ethical standards established in the 1964 Declaration of Helsinki and its later amendments. The datasets from anonymized publicly available publications of the Cancer Imaging Archive data were approved by the local ethics committee and data protection officer.

### Test Set

The test set comprising the international TMTV benchmark group has been previously described (20). In short, it consists of 60 baseline ^18^F-FDG PET/CT scans of 20 patients with follicular lymphoma, 20 with Hodgkin lymphoma, and 20 with diffuse large B-cell lymphoma. The cases were chosen to represent a broad range of ^18^F-FDG distribution patterns, including less common presentations (e.g., focal or diffuse high uptake in the liver and spleen) and were enriched with more challenging cases rather than representative cases of the true prevalence of disease distribution. TMTV was measured by 12 nuclear medicine physicians, each analyzing 20 cases, with each case processed by 3 or 4 readers. Lesions were segmented using an SUV threshold of 4. TMTV ranged from 8 to 2,290 cm^3^ across scans and readers. The reference value for TMTV for each patient was defined as the median TMTV provided by the 3 or 4 readers. An interreader difference in TMTV of less than 10 cm^3^ or 10% was found in 87% of cases in the benchmark dataset. In 13% of cases, discrepancies were caused by differences in lesion inclusion or high-uptake region removal for final TMTV.

### AI Algorithm

Automated segmentation of metabolically active tumor regions was performed using a 3-dimensional convolutional neural network with a 4-level U-Net architecture (26) implemented in MONAI (the medical open network for AI framework) (27). The model inputs were coregistered ^18^F-FDG PET/CT images, resampled to a voxel size of 2.73 × 2.73 × 2.79 mm. Intensities were normalized to a 0–1 range after limiting CT values to −1,024 to 3,072 Hounsfield units and SUVs to 0–100. The network produced a binary segmentation map distinguishing pathologic uptake from background.

The model was trained on 1,500 ^18^F-FDG PET/CT scans of patients with lymphoma, collected from 2 hospitals and 2 publicly available Cancer Imaging Archive datasets. Of these, 1,200 scans were used for training and 300 for validation. Training was performed for 200 epochs on 192 × 192 × 192 voxel patches, with 1 epoch defined as 10,000 samples. The Nesterov-accelerated Adaptive Moment Estimation optimizer (28) (initial learning rate, 5 × 10^−5^; exponential decay factor, 0.985) was used together with a weighted categoric cross-entropy loss (foreground weight, 25.0; background, 1.0). Deep supervision with auxiliary losses (weights of 0.5, 0.25, and 0.125) was applied to improve gradient flow (29). Foreground and background patches were sampled in equal proportions, with adaptive resampling of challenging background regions every 20 epochs.

Inference was performed using a sliding-window approach with 94-voxel overlap to minimize edge artifacts. For voxels with multiple predictions, the value corresponding to the largest effective receptive field was retained. The resulting segmentation was postprocessed using an SUV-based thresholding step—voxels with an SUV of at least 4 and with their nearest local SUV maximum located inside the original AI segmentation were retained as foreground; all others were assigned to background. This produced full-volume, postprocessed segmentations of metabolically active tumor regions without user interaction, enabling direct computation of AI-based TMTVs.

### Statistical Analysis

Agreement between the AI-based TMTVs and the benchmark reference was evaluated using Bland–Altman analysis. According to Boellaard et al. (20), an interreader difference in TMTV of less than 10 cm^3^ or 10% is acceptable. Thus, the proportion of studies fulfilling these criteria were noted.

## RESULTS

**FIGURE 1.**
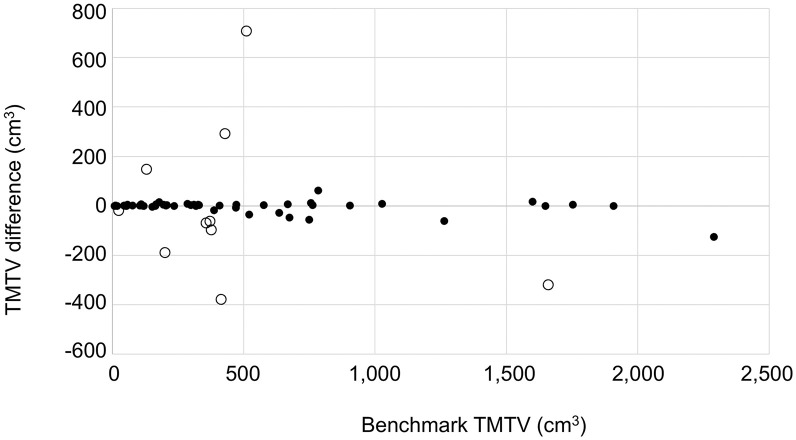
Bland–Altman plot showing difference between AI-derived and benchmark TMTV. Ten cases with largest discrepancies, described in Table 2, are represented by open circles; remaining 50 cases are shown as solid dots.

The AI-derived TMTV ranged from 4 to 2,165 cm³, whereas the benchmark TMTV spanned from 8 to 2,290 cm³. Agreement between AI-derived and benchmark TMTVs is illustrated in the Bland–Altman plot (Fig. 1).

In 50 (83%) of the 60 cases, the AI-derived TMTVs were within 10% or 10 cm³ of the benchmark TMTV, indicating a high level of concordance. The remaining 10 cases, which showed larger discrepancies, are detailed in Table 2. Among these 10 outlier cases, 4 (H15, F02, B16, F05) correspond to patients for whom substantial interreader variability was observed during benchmark TMTV determination, as described by Boellaard (20). In these cases, the AI-derived TMTV either aligned with 1 or 2 of the individual expert readers or fell between the range of values provided by the readers.

**TABLE 2.** Cases with Discrepancies Between AI-Derived and Benchmark TMTV Exceeding Both 10% and 10 cm^3^

Patient No.	Benchmark TMTV (cm^3^)	AI-derived TMTV (cm^3^)	Comment*
Hodgkin lymphoma			
H15	130	279	Individual TMTV estimates: 129 cm^3^, 275 cm^3^, 282 cm^3^
Follicular lymphoma			
F02	376	279	Individual TMTV estimates: 252 cm^3^, 399 cm^3^, 426 cm^3^, 456 cm^3^
F04	428	722	AI included spleen in segmentation
F05	1,658	1,338	Individual TMTV estimates: 567 cm^3^, 1,650 cm^3^, 1,680 cm^3^, 1,706 cm^3^
F08	413	34	AI missed main areas of tumor burden
F13	356	287	AI missed minor parts of tumor bulk
F15	22	4	AI detected tumor but segmented it smaller
DLBCL			
B07	199	11	AI missed widespread bone metastases
B15	371	309	AI missed 1 liver lesion; others segmented accurately
B16	509	1,218	Individual TMTV estimates: 509 cm^3^, 546 cm^3^, 1,223 cm^3^

* Individual TMTV estimates provided by expert readers during benchmark definition process (20).

DLBCL = diffuse large B-cell lymphoma.

**FIGURE 2.**
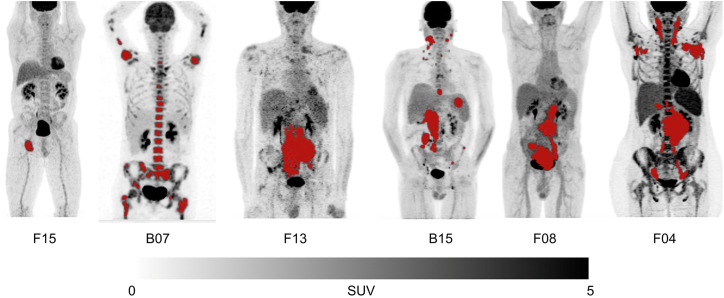
Maximum-intensity-projection images of 6 patients with discrepancies between AI-derived and benchmark TMTV exceeding both 10% and 10 cm^3^. Corresponding details and commentary are provided in Table 2.

The remaining 6 cases, shown in Figure 2, exhibited discrepancies attributable to specific segmentation issues, as annotated in Table 2. These included undersegmentation (e.g., missed bone metastases in B07 or partial tumor volumes in F13 and B15), oversegmentation (e.g., inclusion of the spleen in F04), and differences in tumor boundary delineation.

## DISCUSSION

TMTV is a valuable prognostic and predictive biomarker in both clinical practice and research. However, realization of its full potential is dependent on accurate, reproducible, and efficient methods for tumor segmentation and TMTV calculation. In this study, we evaluated an automated AI-based approach. In 83% (50/60) of the benchmark cases, AI-derived TMTVs were in agreement with the benchmark TMTVs. This is close to the 87% agreement that Boellaard et al. reported between the final benchmark TMTVs and the TMTVs of the 3 or 4 readers involved in the benchmark process (20). In 4 additional cases, the AI-derived segmentations deviated from the benchmark reference but still aligned with at least 1 expert reader involved in the benchmark process.

It is important to note that the benchmark dataset used in this study was not designed for evaluating the clinical performance of segmentation models. Although the TMTVs derived by the AI model showed good agreement with the benchmark TMTVs, this does not necessarily imply that the same individual lesions were segmented by AI and the expert readers. As stated by Boellaard et al., one intended use of the dataset is to evaluate the performance of AI-based segmentations. Furthermore, the benchmark dataset was deliberately enriched with complex and challenging cases that are more difficult than those typically encountered in routine clinical settings (20). This context makes our results particularly encouraging, as they demonstrate robustness of the AI model under demanding conditions.

Generalizability is a crucial aspect of AI model development. Validation on external datasets is essential, as performance on test sets drawn from the same site or scanner as the training data may overestimate the model’s effectiveness.

The strong performance of our AI tool can be partly attributed to the large and diverse training dataset, comprising 1,500 PET/CT studies from patients with various diagnoses across multiple hospitals. This dataset was made possible through publicly available resources such as the Cancer Imaging Archive, highlighting the importance of open-source initiatives in developing generalizable AI tools. However, a limitation of using such datasets is that key clinical metadata, such as diagnosis, age, sex, scan type (baseline or follow-up), were not consistently available because of privacy constraints.

Observed discrepancies between the AI and benchmark segmentations can often be explained by underrepresentation or inconsistencies in the training data. For instance, cases with diffuse splenic involvement or osseous lesions pose challenges not only for AI models but also for human experts, as noted in the benchmark study. These ambiguities underline the need for well-annotated and consistent training data. Furthermore, 6 of the 10 cases with discrepancies shown in Table 2 were patients with follicular lymphoma, a group only represented by 8 patients in the large training set, which is a limitation. Efforts should be made in future training of AI models to include more diverse patients. Further validation of the method is needed in different contexts, such as staging, early or late response assessment, and other diagnoses. The benchmark dataset only included baseline PET/CT scans.

Unlike some existing approaches that rely on fixed SUV thresholds (e.g., SUV of 2.5 or 4 or 41% of SUV_max_), our AI model was trained on manually curated segmentations considered optimal for each case. This allowed for more nuanced learning. Furthermore, postprocessing steps can be added to apply SUV thresholds retrospectively if needed, offering flexibility for different clinical or research requirements.

Our findings demonstrate the feasibility of using an AI-based tool to generate TMTV and segmentations that are, in most cases, clinically usable without manual correction. The potential to reduce tumor segmentation time to near zero represents a significant advantage over current software solutions, which often require manual inclusion or exclusion of regions. As Boellaard et al. have noted, such manual steps not only increase processing time but also introduce interreader variability (20). However, it should be noted that human supervision and correction of the AI model, when needed, are still required.

For now, the AI model is freely available for research use on the RECOMIA platform, where over 75 research groups across 35 countries have already accessed our various AI tools for their own studies.

## CONCLUSION

The AI-based method achieved high concordance with expert-derived TMTVs in a standardized benchmark setting, demonstrating that the AI model performed comparably to human experts in most cases, even in an externally curated dataset deliberately enriched with challenging cases. The AI model’s ability to produce accurate, reproducible segmentations without user interaction could significantly reduce manual workload and interreader variability in lymphoma imaging. However, human supervision is still required to minimize errors.

## DISCLOSURE

This study was supported by the Swedish State under the agreement between the Swedish Government and the Country Councils, the ALF agreement (70380), and Malmö General Hospital Cancer Foundation. Måns Larsson and Olof Enqvist are employees and stockholders of Eigenvision AB, a consultancy company working with research and development in automated image analysis, computer vision, and machine learning. Lars Edenbrandt serves on the board and is a stockholder of Slicevault AB, a company working with streamlining image management for biotech and pharmaceutical companies. No other potential conflict of interest relevant to this article was reported.

## Notes

KEY POINTS **QUESTION:** Can an AI-based method accurately and reproducibly segment TMTV in lymphoma ^18^F-FDG PET/CT scans using a public benchmark dataset? **PERTINENT FINDINGS:** In this validation study, a 3-dimensional U-Net AI model trained on 1,500 lymphoma PET/CT scans was tested on a benchmark dataset of 60 cases segmented by experts. The AI-derived TMTVs were within 10% or 10 cm³ of the benchmark in 83% of cases, demonstrating high concordance with expert readers. **IMPLICATIONS FOR PATIENT CARE:** This AI tool could help physicians image lymphoma more quickly and consistently, which may lead to better treatment planning and less time spent on manual work.
